# Annotations

**Published:** 1910-10-08

**Authors:** 


### Annotations.

### The Committee on Colour-Blindness.

The Board of Trade has recently appointed a Committee on Colour-Blindness, the composition of which has excited the objections of the Imperial Merchant Service Guild and of the editor of the *Ophthalmoscope.* The former objects that the Committee consists mainly of members of the Royal Society and of shipping organisations, whereas the Service Guild itself is quite unrepresented. In 1892 it was on the Royal Society's advice that the Board of Trade adopted Hohngren's wool-tests officially for the detection of colour-blindness; and it is regarded as improper that, with the Royal Society as it were on trial, the jury should consist so largely of its members. The protest of the *Ophthalmoscope* is based upon other grounds, but they are equally important. Dr. Edridge Green advised the Board of Trade against the adoption of the Royal Society's recommendations when the tests were originally discussed. He has worked hard and persistently at this subject ever since, and has witnessed the gradual recognition of several of his principles. To leave him out of the Committee is an error both of judgment and tactics, and the omission is characterised by the *Ophthalmoscope* as indeed a glaring one. Further, it is suggested that, failing the appointment of Dr. Edridge Green to the Committee forthwith, the matter should be ventilated in the House of Commons. While this course would probably do no good and might provoke political recrimination, it is certainly true that the Committee on Colour-Blindness has been unfortunately constituted.

### Trachoma in Queensland.

Serious as is the prevalence of trachoma in Egypt, it would almost seem that Western Queensland is in nearly as bad a state, on the testimony of Dr. E. T. Smith, who is endeavouring to arouse Australian public opinion in the matter. An ophthalmic surgeon who two years ago toured that district on behalf of the Government reports that 90 per cent. of school children have "more or less unhealthy lids," and that 20 per cent. have some permanent impairment of vision from trachoma. It will be credited only with difficulty—and with indignation—that the authorities to whom this report was made have done practically nothing to abolish this reproach, and that of their own inspector's twelve recommendations two only have been carried out—even those but partially. School teachers have been supplied with pots of ointment, and a few fly-proof blinds have been supplied to schools, and that is about all that has been done. Dr. Temple Smith well remarks that a day may come when Australian freedom will depend on keen eyesight behind the rifle to repel invaders. If 20 per cent. of the young men cannot see their rifle-sights owing to preventable disease, the fault will lie with those who have neglected to do for their own people what England is starting to do for the Egyptian fellaheen. The suggestion is advanced that the travelling hospital system which has been instituted in Egypt may be the best, after adaptation, to meet the requirements of Western Queensland. In each township or other centre visited by the flying ophthalmic hospital the local nursing staff, local hospital, and local doctor could be utilised. In protesting so emphatically against the very grave deficiencies of a Government too apathetic to protect the eyesight of the coming generation, the author of the article in the *Australasian Medical Gazette*, from which are taken the above facts, is doing his country a real service. We wish him well most heartily in his hygienic campaign.

### Osteopathy.

We have had several letters dealing with what is usually, but entirely erroneously, known as "bone-setting"; two of these letters, being more suitable for publication than the rest, we print in our correspondence columns. Perhaps it is worth while to remind our correspondents that personalities are much to be deprecated in a discussion of this kind; it is the method, and not the man who employs it, that needs advertisement, if it is a helpful aid to treatment, and criticism and condemnation if it is the reverse. What we want, and what we are willing to publish when we get them, are details of actual cases, with the records of treatment, and, what is specially important, of the condition some time after treatment. Dr. Garrard, who is in a position to judge with knowledge and criticise with a full cognisance of the responsibility that a professional man of standing incurs by taking his patients to an outside consultant, gives us two cases; but, unfortunately, he does not give them in detail, and there is no means of tracing their after history. What we should have liked to have before us are the actual histories of these cases, the names of the "eminent consultants" who attended them, the radiographic and other evidence of the lesions which existed and baffled ordinary treatment, and many other points. If correspondents are able to furnish us with these data we shall be in a position to deal more fully with the whole matter. Many of the methods used by "bone-setters" in this country and in America are well known to German, French, and Italian orthopædists, though they are not usually described in text-books, for one reason because they cannot be fully described without the aid of elaborate diagrams, and for another because they are reckoned unsuitable for practitioners who have not had a wide and extended experience. Unfortunately they are not well known in this country, and there is a tendency—how far justifiable we will not for the moment presume to judge—to regard them as unnecessary and dangerous aids. We hope to return to this subject in a future issue and to deal with it at length. In the meantime we shall be glad to have reliable evidence and records of cases, and we would urge on correspondents the desirability of having such lesions as have been treated by "bone-setters" adequately and carefully skiagraphed. It is only in that way that corroborative evidence can be obtained for or against the claims of osteopathy.

